# Comparing Medication Non-adherence in Cardiovascular Disease Patients at Public and Private Hospitals in Peshawar: A Cross-Sectional Study of Prevalence and Contributing Factors

**DOI:** 10.7759/cureus.36345

**Published:** 2023-03-19

**Authors:** Azhar Saeed, Qazi Kamran Amin, Rida Saeed, Zaland A Yousafzai

**Affiliations:** 1 Internal Medicine, Stockport National Health Service (NHS) Foundation Trust, Manchester, GBR; 2 Medicine, Rehman Medical Institute, Peshawar, PAK; 3 Internal Medicine, Mercy Teaching Hospital, Peshawar, PAK

**Keywords:** pilot projects, confidence intervals, health expenditures, financial stress, developing countries, cardiovascular diseases, oral medicines, medicines, adherence, non-adherence

## Abstract

Introduction

The incidence of cardiovascular diseases continues to increase, becoming one of the leading causes of mortality globally. The proper use of medication can greatly reduce the death rate by slowing the progression of the disease. Yet, many patients struggle with following their medication regimen due to various reasons. Effective treatment management relies on patients' self-care and understanding of their illness and medications, which can impact their adherence to taking their prescribed drugs. The objective of the study was to determine the prevalence of medication non-compliance among patients in two public and private tertiary care hospitals in Peshawar and to identify the factors that contribute to this behavior.

Material and methods

A comparative cross-sectional study design was employed for the research. The study was conducted at Hayatabad Medical Complex and Rehman Medical Institute in Peshawar, as these two hospitals provide care for a significant proportion of cardiovascular disease patients in the area. To assess adherence, a quantitative scale was devised with scores of 8 considered high adherence, scores between 6 and 7 considered medium adherence, and scores below 6 considered low adherence. The factors impacting medication non-adherence were analyzed using a self-administered questionnaire, which was developed following a preliminary study conducted at both hospitals.

Results

In total, 168 eligible patients from the two hospitals were given the questionnaire. Out of these patients, 107 (63.7%) were male, and 61 (36.3%) were female, with ages ranging from 19 to 84 and a mean age of 55.33. The level of medication adherence was calculated among the participants, with 20.2% reporting high adherence, 22.6% reporting medium adherence, and 57.1% reporting low adherence. The results showed that monthly income (p = 0.006), the presence of co-morbidities (p = 0.002), and the fear of addiction to medication (p = 0.048) were the main factors influencing medication adherence. In regression analysis with high adherence as the reference category and a 95% confidence interval, hospital affiliation was found to be significantly associated with adherence levels.

Conclusions

The study found that medication adherence among cardiovascular disease patients in private tertiary care hospitals is generally high. However, the level of adherence was seen to be impacted by the patient's monthly income. In light of this, the government should implement programs to reduce the cost of healthcare provision and increase affordability for patients.

## Introduction

Background

Cardiovascular diseases, as the name indicates, are diseases of the heart and blood vessels. The four main types are coronary heart disease, stroke, peripheral vascular disease, and aortic disease [1]. Medication adherence is defined as the regular and continuous taking of prescribed medicine by the patient. C. Everett Koop, MD, stated that "Drugs don't work in patients who don't take them" [2].

A study conducted in Quetta, Pakistan, showed that there is a lack of education among patients regarding the benefits of medication adherence [3]. The mortality rate for cardiovascular diseases is expected to increase in developing countries such as Pakistan over the next couple of decades due to high life expectancy and changes in lifestyle. In fact, cardiovascular diseases have reached a state of epidemic in many developing countries around the world [4]. Studies done in the United States have shown that more than 60% of cardiovascular disease patients are reported to be non-adherent, which is a growing cause of concern [5]. The factors affecting adherence may be physician- or patient-related and can lead to significant expenditure on the healthcare system, estimated at roughly $396-$792 million [6]. By addressing the problem of non-adherence, a lot of unnecessary spending on healthcare can be avoided [7]. Non-adherence is a problem not only in developing countries but also in developed countries. It is estimated that each year, 125,000 deaths from cardiovascular diseases in the United States are caused by non-adherence [8]. Identifying non-adherence can help in successfully treating the condition. If left unidentified, non-adherence can lead physicians to intensify the drug regimen, which in turn can lead to undesired outcomes and a burden on the economic state [9,10].

Physicians must understand that poor medication adherence is one of the reasons for below par clinical benefits. The World Health Organization (WHO) states that increasing medication adherence has a greater impact on health than improving specific treatments [11]. Therefore, physicians should identify strategies to improve medication adherence within the limits of their practice to enhance therapeutic outcomes. This approach should be carried out with the support and involvement of all individuals involved in medication use [12]. High medication adherence is associated with a decrease in emergency department visits, a decrease in hospitalization days, and a decrease in a person's pharmacy spending [13].

Medication non-adherence has been a growing concern in our community as many people do not adhere to the drug regimen prescribed by their doctor, leading to further complications of their disease. As a developing country, the financial burden increases on individuals and the government to provide proper health services. This cannot be done until patients observe medication adherence.

There have been no previous studies conducted in this region with this scope. Our study aims to calculate the frequency of medication non-adherence and identify various factors such as demographics; illness; and patient-, physician-, and medication-related factors associated with non-adherence.

The study aims to assess the incidence of non-compliance with medication among patients suffering from cardiovascular disease who seek treatment at both public and private tertiary care hospitals. It also endeavors to uncover the factors contributing to medication non-adherence among these patients in both types of healthcare facilities.

## Materials and methods

Study design

This study utilized a comparative cross-sectional design and was conducted during the months of April and May in 2016. The sample size was calculated using the WHO sample calculator and was found to be 168, divided equally between both public and private tertiary care hospitals in Peshawar. The frequency of medication non-adherence was compared in both hospitals, and various factors affecting medication adherence were calculated.

Setting

Data was collected from the outpatient department of two tertiary care hospitals in Peshawar: Hayatabad Medical Complex, a public sector tertiary care hospital located in Hayatabad, Peshawar, where around 150 patients visit the cardiology outpatient department daily, and Rehman Medical Institute, a private sector tertiary care hospital located in Hayatabad, Peshawar, where around 60 patients visit the cardiology outpatient department daily.

Participants

Study participants were identified based on clearly defined selection criteria. Patients who have been using cardiovascular disease medication for more than six months were included, while patients who were being supported by any government or private organizations, have dementia and other neuronal degenerative problems, and were dependent on others for taking their medications were excluded.

Study size

A study conducted in India showed medication non-adherence among chronic diseases in about 11.76% of the patients, which was used as the prevalence to calculate the sample size from the WHO calculator and was found to be 168 (84 per hospital).

Variables

The variables that have a major impact on medication adherence were included in the analysis. These variables included marital status, income per month, education status, drug regimen, co-morbidities, alternate remedies, and confidence in the doctor. These variables were correlated with the level of adherence found among patients in both hospitals. The factors that affect medication adherence were divided into illness, medication, and physician-related factors.

Data collection and analysis

A self-structured questionnaire was used to gather data regarding all variables, and the data was analyzed using Statistical Package for Social Sciences (SPSS) version 16 (IBM SPSS Statistics, Armonk, NY). There was no difference in characteristics between the patients selected from the private and public sector tertiary care hospitals.

Quantitative variables

A quantitative scale was devised in which 8 would be considered high adherence, 6-8 medium adherence, and <6 low adherence. The association of adherence with different variables such as monthly income, literacy, number of drugs used, and co-morbidities in both the private and public tertiary care hospitals selected was also analyzed.

Statistical methods

The frequency of medication adherence was calculated using percentages. To compare the levels of adherence between the two types of hospitals, crosstabs were used, and the results were displayed in a pie chart format. A chi-square test was performed to determine the relationship between the level of adherence and various factors that may affect it. Additionally, multinomial logistic regression was employed to examine the significance of the association between medication adherence and other variables, with high adherence serving as the reference category.

## Results

In total, 168 patients from the two hospitals who met the eligibility criteria were surveyed using questionnaires. Out of these participants, 107 (63.7%) were male, while 61 (36.3%) were female. The age range of the subjects was from 19 to 84 years old, with an average age of 55.33. Table 1 displays the percentage distribution of all variables studied, and Table 2 illustrates the distribution of gender with respect to medication adherence.

**Table 1:** Various variables described in both hospitals with confidence intervals (CI)

	Hayatabad Medical Complex				Rehman Medical Institute			
	Number	Percent	95.0% Lower CI	95.0% Upper CI	Number	Percent	95.0% Lower CI	95.0% Upper CI
Male	62	36.9%	29.9%	44.4%	45	26.8%	20.5%	33.8%
Female	22	13.1%	8.6%	18.8%	39	23.2%	17.3%	30.0%
Married	81	48.2%	40.7%	55.7%	82	48.8%	41.3%	56.3%
Unmarried	3	1.8%	0.5%	4.7%	2	1.2%	0.2%	3.8%
<10,000	11	6.5%	3.5%	11.0%	11	6.5%	3.5%	11.0%
10,000-20,000	22	13.1%	8.6%	18.8%	16	9.5%	5.8%	14.7%
20,000-40,000	34	20.2%	14.7%	26.8%	33	19.6%	14.2%	26.1%
40,000-80,000	14	8.3%	4.9%	13.2%	14	8.3%	4.9%	13.2%
>80,000	3	1.8%	0.5%	4.7%	10	6.0%	3.1%	10.3%
Illiterate	43	25.6%	19.5%	32.6%	45	26.8%	20.5%	33.8%
Primary	13	7.7%	4.4%	12.5%	18	10.7%	6.7%	16.1%
Intermediate	17	10.1%	6.2%	15.4%	13	7.7%	4.4%	12.5%
Undergraduate	10	6.0%	3.1%	10.3%	6	3.6%	1.5%	7.2%
Postgraduate	1	0.6%	0.1%	2.7%	2	1.2%	0.2%	3.8%

**Table 2:** Gender-wise distribution of patients on the basis of level of adherence

			Gender of the Responder	
			Male	Female
Level of Adherence	Low Adherence	Count (% Within the Level of Adherence)	64 (66.7%)	32 (33.3%)
	Medium Adherence	Count (% Within the Level of Adherence)	25 (65.8%)	13 (34.2%)
	High Adherence	Count (% Within the Level of Adherence)	18 (52.9%)	16 (47.1%)

We hypothesized that there is a difference in the medication adherence of cardiovascular drugs among patients coming to public and private sector tertiary care hospitals, which was confirmed, and the null hypothesis was rejected.

**Figure 1:**
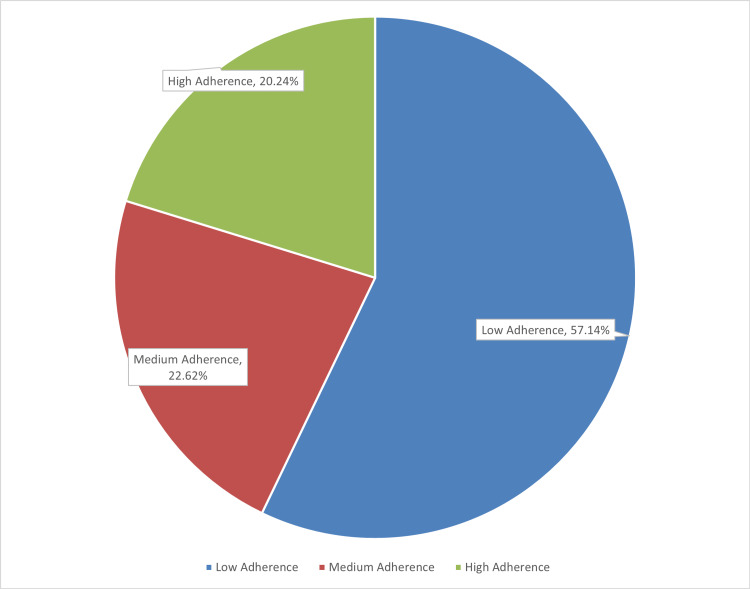
Pie chart showing the overall percentage of adherence level in all patients

The level of adherence was calculated among both hospitals and is described in Table 3. The level of adherence was cross-tabulated with both hospitals, and the strength of association was calculated using a chi-square test (α = 0.05), which was found to be highly significant (p = 0.000). This suggests that the hospitals are affecting the level of medication adherence among patients as given in Figure 1.

**Table 3:** Level of adherence in both hospitals

			Level of Adherence		
			Low Adherence	Medium Adherence	High Adherence
Name of the Hospital	Hayatabad Medical Complex	Count (% Within the Name of the Hospital)	63 (75.0%)	17 (20.2%)	4 (4.8%)
	Rehman Medical Institute	Count (% Within the Name of the Hospital)	33 (39.3%)	21 (25.0%)	30 (35.7%)

Chi-square tests were carried out to find the strength of association between the level of adherence and the variables. Among the variables tested, income per month was found to be statistically significant (p = 0.006) in the private tertiary care hospital. Co-morbidities (other diseases) and addiction to medication were found statistically significant with p = 0.002 and 0.048, respectively, in the public tertiary care hospital.

A multinomial regression analysis was performed to determine if there was a significant correlation between the level of medication adherence and factors that impact it. High adherence was designated as the reference category, and a 95% confidence interval was used, as presented in Table 4.

**Table 4:** Multinomial regression analysis ^a^The reference category is high adherence df: degrees of freedom

Level of Adherence^a^		Standard Error	df	Significance	Exp(B)	95% Confidence Interval for Exp(B)	
						Lower Bound	Upper Bound
Low Adherence	Intercept	1.316	1	0.000			
	Hospital	0.585	1	0.000	0.068	0.022	0.215
	Education	0.207	1	0.375	0.833	0.555	1.248
Medium Adherence	Intercept	1.395	1	0.023			
	Hospital	0.629	1	0.004	0.164	0.048	0.564
	Education	0.214	1	0.986	1.004	0.660	1.528

## Discussion

According to this study, the overall medication adherence was observed as low adherence, 57.1%; medium adherence, 22.6%; and high adherence, 20.2%. The patients of private tertiary care hospital showed a high adherence of 35% compared to 4.8% in the public tertiary care hospital. Income per month was the main variable, which was significant in this regard.

Few studies have been conducted in Pakistan regarding medication adherence among cardiovascular disease patients. A study conducted in Abbottabad, Pakistan, revealed that 68.14% of patients were having low medication adherence. This low adherence was found to be significantly associated with gender and socioeconomic status [14]. Similarly, another study was conducted on 460 patients at the Aga Khan University Hospital (AKUH) and National Institute of Cardiovascular Diseases, Karachi, from September 2005 to May 2006; 77% of the patients were found to have high adherence, and the significant factors associated with it were the number of drugs taken by the patient and the regularity of medication [15].

An Ethiopian study, conducted on 384 patients, revealed that 64.6% of the patients were adherent to their treatment. Gender, knowledge about treatment, distance from the hospital, and co-morbidities were significantly associated factors [16]. Another study conducted in Uzbekistan, on 209 patients, revealed that 36.8% of the patients had a low level of adherence; knowledge regarding the disease was significantly associated with low medication adherence [17]. A study conducted in India in 2022 revealed that medication adherence was 20.83%, 28.37%, and 32% in hypertension (HTN), congestive cardiac failure (CCF), and ischemic heart disease (IHD) patients, respectively [18]. A study conducted in rural India on 280 patients revealed that about 32% of the patients had low adherence [19].

Additionally, recent studies have found that factors such as language barriers, lack of social support, and cultural beliefs also play a role in medication non-adherence among cardiovascular disease patients [20,21]. A study conducted in the United States found that Hispanic patients had lower adherence rates compared to non-Hispanic patients, due to language barriers and the lack of cultural competence among healthcare providers [22]. Another study in the United Kingdom found that patients from South Asian backgrounds had lower adherence rates due to cultural beliefs and the lack of social support [23].

Thus, it appears that medication adherence among cardiovascular disease patients generally remains the same across different countries belonging to the Asian region among which income and co-morbidities tend to be significantly associated with adherence.

Limitations

This study is unable to establish a temporal sequence of events. There may also be recall bias due to the use of self-reported questionnaires.

Generalizability

The findings of this study can be extrapolated to other tertiary care hospitals within the same province or country, as the patient demographic and healthcare services tend to exhibit similarities.

Recommendation

It is necessary for the government to consider subsidizing healthcare costs to make healthcare services more affordable to low-income patients and the healthcare providers to take a patient-centered approach to healthcare delivery, which includes tailoring treatment plans to meet patients' individual needs and addressing their fears and concerns regarding medication use. By implementing these measures, we can improve medication adherence, enhance patient outcomes, and reduce the burden of cardiovascular disease on individuals and the society as a whole.

## Conclusions

The findings of this study indicate that medication adherence among cardiovascular disease patients is higher in private sector tertiary care hospitals than in the public sector ones. Income, co-morbidities, and the fear of addiction were found to be significant factors affecting medication adherence. The government should consider implementing programs to subsidize the cost of healthcare to address the issue of low adherence among low-income patients.
